# Quantifying in vivo scaphoid, lunate, and capitate kinematics using four-dimensional computed tomography

**DOI:** 10.1007/s00256-020-03543-4

**Published:** 2020-07-30

**Authors:** Michelle Brinkhorst, Mahyar Foumani, Joost van Rosmalen, Ruud Selles, Steven Hovius, Simon Strackee, Geert Streekstra

**Affiliations:** 1 Department of Plastic, Reconstructive and Hand Surgery, Erasmus MC, University Medical Center Rotterdam, Rotterdam, the Netherlands; 2 Department of Plastic, Reconstructive and Hand Surgery, Martini Hospital, Groningen, the Netherlands; 3 Department of Biostatistics, Erasmus MC, University Medical Center Rotterdam, Rotterdam, the Netherlands; 4 Department of Rehabilitation Medicine, Erasmus MC, University Medical Center Rotterdam, Rotterdam, the Netherlands; 5 Xpert Clinic, Hand and Wrist Clinic, Amsterdam, the Netherlands; 6 Department of Plastic, Reconstructive and Hand Surgery, Radboudumc, Nijmegen, the Netherlands; 7 Department of Plastic, Reconstructive and Hand Surgery, Amsterdam UMC, University of Amsterdam, Amsterdam, the Netherlands; 8 Department of Biomedical Engineering and Physics, Amsterdam UMC, University of Amsterdam, Amsterdam, the Netherlands; 9 Department of Radiology and Nuclear Medicine, Amsterdam UMC, University of Amsterdam, Amsterdam, the Netherlands

**Keywords:** Wrist joint, In vivo kinematics, Carpal kinematics, Dynamic, Four-dimensional CT imaging

## Abstract

**Objective:**

We aimed to establish a quantitative description of motion patterns and establish test-retest reliability of the four-dimensional CT when quantifying in vivo kinematics of the scaphoid, lunate, and capitate.

**Materials and methods:**

We assessed in vivo kinematics of both wrists of 20 healthy volunteers (11 men and 9 women) between the ages of 20 and 40 years. All volunteers performed active flexion-extension and radial-ulnar deviation with both wrists. To test for reliability, one motion cycle was rescanned for both wrists approximately 15 min after the first scan. The coefficient of multiple correlation was used to analyze reliability. When two motion patterns are similar, the coefficient of multiple correlation tends towards 1, whereas in dissimilar motion patterns, it tends towards 0. The root mean square deviation was used to analyze the total motion patterns variability between the two scans.

**Results:**

Overall, mean or median coefficient of multiple correlations were higher than 0.86. The root mean square deviations were low and ranged from 1.17° to 4.29°.

**Conclusion:**

This innovative non-invasive imaging technique can reliably describe in vivo carpal kinematics of uninjured wrists in healthy individuals. It provides us with a better understanding and reference values of carpal kinematics of the scaphoid, lunate, and capitate.

**Electronic supplementary material:**

The online version of this article (10.1007/s00256-020-03543-4) contains supplementary material, which is available to authorized users.

## Introduction

The wrist is one of the most complex joints in the human body. Each carpal bone can perform multiplanar movements. To functionally stabilize the wrist, numerous strong ligaments interconnect wrist bones to surrounding structures, allowing them to function cohesively. By analyzing motion patterns of carpal bones quantitatively, we expect to be able to differentiate between normal and abnormal wrist kinematics which occur after ligament disruption [1, 2].

Four-dimensional computed tomography (4D-CT) was introduced for the acquisition of dynamic 3D images of a moving wrist joint [3–7]. It yields a series of time-resolved 3D images, which allows studying individual wrist bone movements in a non-invasive way. The strengths of the 4D-CT method compared with other imaging methods for measurement of carpal kinematics, like 4D-RX [8, 9], is that it fits in the clinical workflow and that it needs limited acquisition time.

Until today, studies evaluated cadaveric wrists to describe radiocarpal kinematics [4, 5, 7, 10]. In addition, most studies did not quantitatively analyze the motion patterns of the carpal bones. For example, Zhao et al. [11] showed that the accuracy of this technique during in vitro simulated wrist movement was comparable with other image-based kinematic techniques like fluoroscopy, static biplane radiography, and CT. However, the latter measurements were performed in vitro. Consequently, quantitative 4D-CT-based data describing the in vivo motion patterns of the wrist joint and the associated reliability of 4D-CT are lacking. Such data is essential for comparison of motion patterns of the wrist of pathological wrists with those of healthy volunteers. Therefore, the purpose of this study was to establish a quantitative description of motion patterns and associated reliability of a 4D-CT analysis of in vivo scaphoid, lunate, and capitate kinematics in healthy adults.

## Materials and methods

### Participants

Subjects were included if they had no history of wrist injuries or heavy manual work and were aged between 20 and 40 years. Both wrists of 20 healthy volunteers (11 males and 9 females) with a mean age of 27 years (SD 4) were studied. This study was approved by the Medical Ethical Committee of our institute, and informed consent was obtained from each subject.

### Wrist motion guiding device

**Fig. 1:**
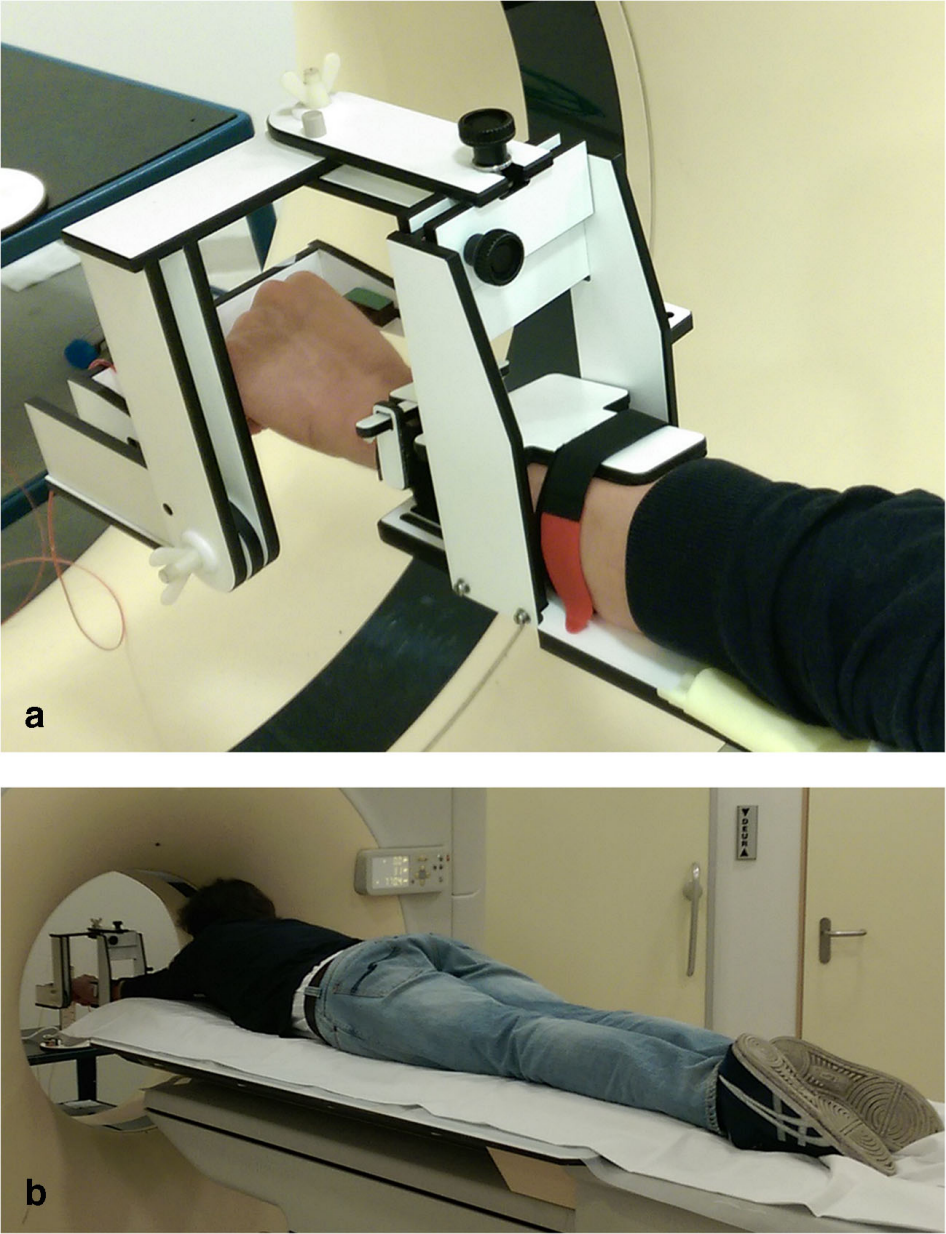
**a** Custom-made device to guide the hand and wrist during the 4D-CT recordings. **b** Positioning of a participant during 4D-CT recordings

A custom-made motion guiding device was designed, implemented, and, subsequently, used to minimize inter-individual differences in positioning the arm during scanning and guiding the hand and wrist during the 4D-CT recordings (Fig. 1a). Participants were put in a prone position with one arm 180° abducted (Fig. 1b). The forearm was placed in the motion guiding device with the elbow extended and the forearm fixed to a framework. Metacarpals were placed in a natural position on a handlebar that is attached to the framework. Subjects were instructed to lightly grab the handlebar while actively performing maximum flexion-extension motion or radial-ulnar deviation with their wrist.

### 4D-CT imaging protocol

A Brilliance 64-slice CT scanner (Philips Medical System; Best; The Netherlands) was used for 3D and 4D-CT acquisitions. To reconstruct the geometry of the carpal bones, a static CT image of the wrist was taken with the wrist in a neutral position. Static CT scans of the wrists were made at 120 kV and 75 mAs. The CT images were checked for gross pathology of the wrist, including possible internal derangement of carpal bones.

Subsequently, one flexion-extension or radial-ulnar deviation motion cycle was imaged in approximately 10 s with a 4D-CT protocol. Collimation of the 4D scans was 64 × 0.625 mm, resulting in an axial field of view of 4 cm. Applied tube voltage and charge per time frame were 120 kV and 15 mAs, respectively. Voxel size of the static scan was 0.33 × 0.33 × 0.33 mm. Voxel size of a 4D frame was 0.49 × 0.49 × 0.62 mm. Sampling frequency was 2.5 Hz (every 0.4 s), and the exposure for each time frame time was 0.27 s. Thirty different 3D time frames per complete motion cycle were collected with each 4D-CT scan.

Randomization was used to decide which wrist was scanned first. After both wrists were scanned, subjects were asked to leave the room. Then, to establish test-retest reliability, the subject was asked back, and one motion cycle was rescanned for each wrist approximately 15 min after the first scan. Randomization was also used to decide which motion cycle (flexion-extension or radial-ulnar deviation) of a wrist was rescanned. When the right wrist was randomly assigned to repeat flexion-extension motion, the left wrist repeated radial-ulnar deviation motion. A total of 10 dominant wrists and 10 non-dominant wrists were rescanned for the flexion-extension motion cycle, and a total of 10 dominant wrists and 10 non-dominant wrists were rescanned for the radial-ulnar deviation motion cycle to establish reliability. Data was saved and stored anonymously.

### Segmentation and estimation of translation and rotation of the carpal bones

After all anonymized data was collected, segmentation of the images was performed. The method of segmentation and estimation of translation and rotation of the radius and carpal bones used in the present study was first introduced by Carelsen [8] and Foumani [9]. In short, segmentation of the carpal bones and radius from the CT image was semi-automatically performed by a region-growing algorithm using custom-made software [12]. Only the static scan was segmented. Each scan consisted of 300–350 slices with 0.33-mm distance in between. To estimate rotation and translation of the individual bones relative to the neutral position, the segmented radius and carpal bones were registered to the corresponding bones in the volumes of the dynamic scan.

### Kinematics

**Fig. 2:**
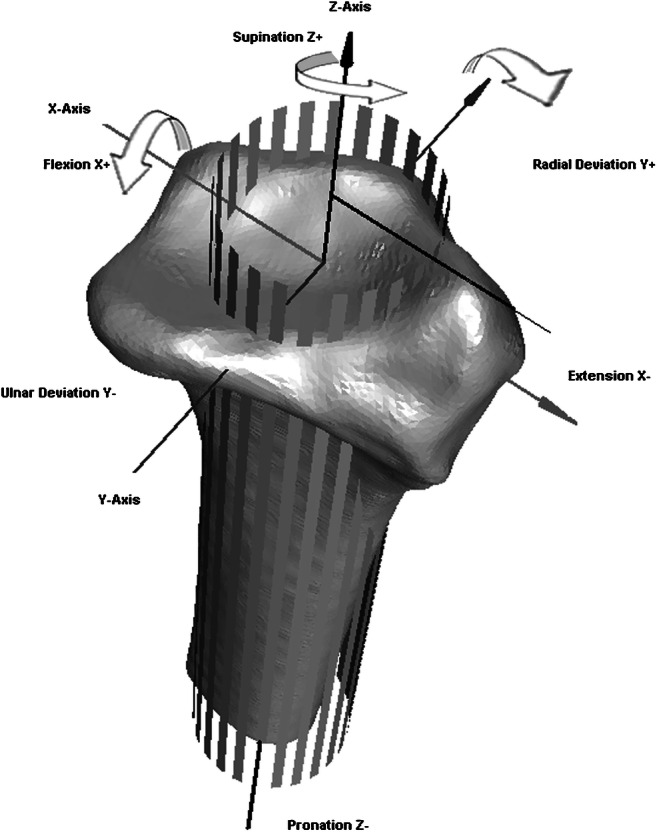
Anteroradial view of a right radius. Repositioning of the carpal bones is expressed in terms of an anatomically based coordinate system for the radius (9). The X-axis goes straight through the distal radioulnar joint towards the radial styloid and rotation around the X-axis indicates flexion (+) or extension (−). The Y-axis is perpendicular to the X-axis, and rotation around the Y-axis indicates ulnar deviation (−) or radial deviation (+). The Z-axis is perpendicular to the X- and Y-axis and is based on the longitudinal axis of the radius. The rotation around the Z-axis indicates supination (+) or pronation (−). Reprinted from Journal of Biomechanics, 42, Foumani, M., Strackee, S.D., Jonges, R., Blankevoort, L., Zwinderman, A.H., Carelsen, B., Streekstra, G.J., In-vivo three-dimensional carpal bone kinematics during flexion-extension and radio-ulnar deviation of the wrist: dynamic motion versus step-wise static wrist positions, 2664–2671, Copyright (2009), with permission from Elsevier

The scaphoid, lunate, and capitate were analyzed to describe radiocarpal kinematics, since those are the key players in wrist joint function. To express various motion parameters of individual carpal bones, an anatomically based radial coordinate system, described by Kobayashi et al. [13], was used (Fig. 2). Coordinate axes of the scaphoid, lunate, and capitate were defined as being parallel to this radial coordinate system with the wrist in a neutral position. Furthermore, wrist motion was defined as the rotation of the capitate relative to the radius [14, 15]. Subsequently, flexion was defined as “+” and extension as a “−” rotation around the X-axis, radial deviation as a “+” and ulnar deviation as a “−” rotation around the Y-axis, and pronation as a “−” and supination as a “+” rotation around the Z-axis.

Only rotational parameters of the scaphoid, lunate, and capitate were evaluated in this study since previous studies showed that translations of carpal bones were marginal [15–17]. To describe the rotational parameters, the helical axis and attitude vector were determined [18]. The helical axis, which is essentially the axis of rotation of a carpal bone during motion, was estimated for each time frame in a 4D-CT sequence from the previously determined neutral position and the position during motion. Subsequently, the attitude vector was defined by a multiplication of the amount of rotation around the helical axis and the unit vector of pointing in the direction of the helical axis. Eventually, rotation parameters in X-, Y-, and Z-direction were estimated based on components of the attitude vector in the radial coordinate system.

### Statistical analysis

All complete motion cycles were linearly interpolated to obtain data at every 1° of radial wrist motion, in which global wrist motion was defined as degrees of capitate rotation around the X-axis during flexion-extension and Y-axis during radial-ulnar deviation. Primary outcome variables per carpal bone were defined as the X, Y, and Z rotational components per 1° of global wrist motion.

Test-retest reliability was calculated with the coefficient of multiple correlation (CMC), first described by Kadaba et al. [19], and the root mean square deviation (RMSD). The CMC was calculated to evaluate the similarity between two motion patterns. When two motion patterns are similar, the CMC tends towards 1, whereas in dissimilar waveforms, it tends towards 0. The CMC is defined mathematically as

1 $$ {CMC}_i=\sqrt{\frac{\sum_{t=1}^2\sum \limits_a{\left({Y}_{iat}-{\overline{Y}}_{it}\right)}^2/A}{\sum_{t=1}^2\sum \limits_a{\left({Y}_{iat}-{\overline{Y}}_i\right)}^2/\left(2A-1\right)}}, $$

where CMC_*i*_ s the CMC value of test subject *i*, *Y*_iat_ is the helical rotation of test subject *i* at motion cycle *t* (*t* = 1 or *t* = 2) with a global wrist motion of *a* degrees, and *A* denotes the number of wrist motion angles in the summation. ${\overline{Y}}_{it}$ is defined as the average of the helical rotations for subject *i* at motion cycle *t* (i.e., ${\overline{Y}}_{it}=\frac{1}{A}\sum \limits_a{Y}_{iat}$), and ${\overline{Y}}_i$ is the average of all helical rotations for subject *i* (i.e., ${\overline{Y}}_i=\frac{1}{2A}{\sum}_{t=1}^2\sum \limits_a{Y}_{iat}$). In all summations with respect to the global wrist motion, only angles are included that were observed at both motion cycles for that specific subject.

The CMC value is related to several aspects of the data and the data collection procedure. By removing the vertical offset, inconsistencies in wrist placement during scanning and segmentation of the individual bones is minimalized. Therefore, individual CMC values were calculated with and without correction for vertical offset between motion cycles [20]. The correction for offset consisted of subtracting ${\overline{Y}}_{it}$ from all observed *Y*_*iat*_ before calculating the result of Eq. 1. The mean and standard deviation of the individual CMC values, with and without correction for offset, were reported for all rotations per carpal bone. If the CMC was not a real number for one or more test subjects, due to a negative value inside the square root, the median and interquartile range were reported instead.

To analyze total waveform variability present between test and retest, the RMSD was used. RMSD represents the sample standard deviation of the differences in the measured angles between the first and the repeated motion. It is calculated by taking the root of the mean squared difference between the two motion cycles. Statistical analyses were performed using SPSS version 21.

## Results

### Check for wrist pathology

No pathology was observed in the static CT scans of the wrists. Consequently, the total of 20 wrists could be used for further evaluation.

### Carpal kinematics

**Fig. 3:**
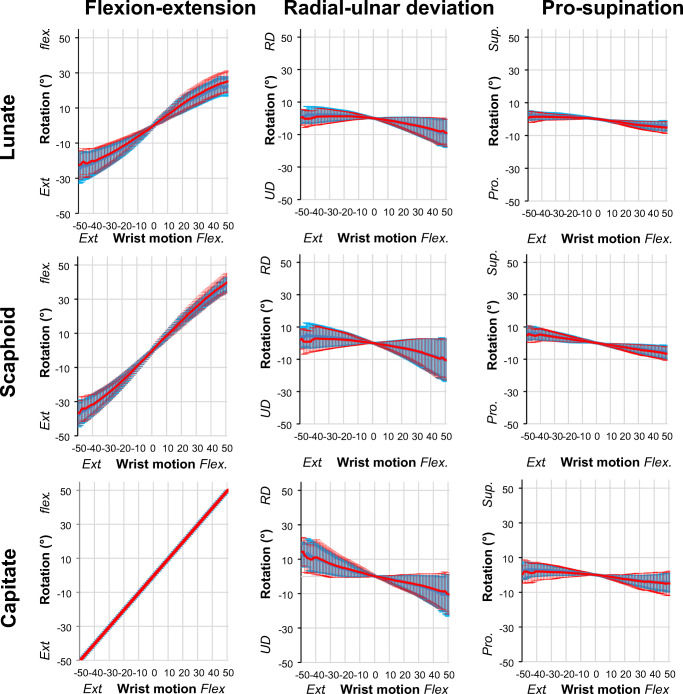
Comparison of the first scan (light blue) and the repeated scan (red) motion patterns in healthy volunteers during flexion-extension of the wrist. Mean and standard deviations of flexion-extension, radial-ulnar deviation and pro-supination of the lunate, scaphoid, and capitate are plotted for every degree of wrist motion. Flexion-extension motion of the capitate is presented as a straight red line as the flexion-extension motion of the capitate was used to define the global wrist motion. Ext, extension; Flex, flexion; Pro, pronation; Sup, supination; UD, ulnar deviation; RD, radial deviation

**Fig. 4:**
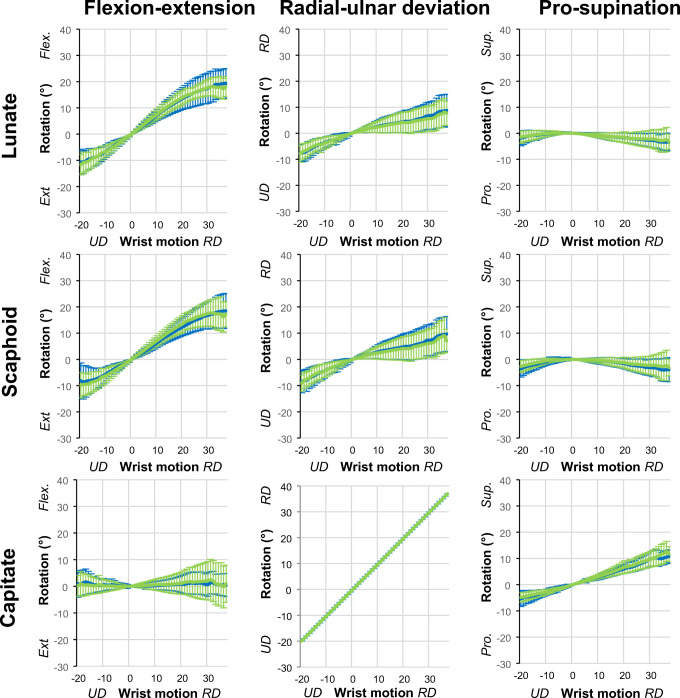
Comparison of the first scan (blue) and the repeated scan (light green) motion patterns in healthy volunteers during radial-ulnar deviation of the wrist. Mean and standard deviations of flexion-extension, radial-ulnar deviation, and pro-supination of the lunate, scaphoid, and capitate are plotted for every degree of wrist motion. Radial-ulnar deviation motion of the capitate is presented as a straight green line as the flexion-extension motion of the capitate was used to define the global wrist motion. Ext, extension; Flex, flexion; Pro, pronation; Sup, supination; UD, ulnar deviation; RD, radial deviation

The lunate and scaphoid both flex when the wrist is flexed, and both extend when the wrist is extended (Fig. 3, Video 1). The lunate, scaphoid, and capitate deviate ulnar during flexion of the wrist and all deviate radial when the wrist is extended. During radial-ulnar deviation of the wrist, the lunate and scaphoid extend when the wrist is ulnar deviated and flex when the wrist is radial deviated (Fig. 4, Video 2).

### Test-retest reliability

Overall, median or mean CMC were larger than 0.77 except for the flexion-extension pattern of the capitate during radial-ulnar deviation of the wrist (Table 1). After the offset was removed, median or mean CMC was improved (range 0.86–1). RMSDs during flexion and extension of the wrist were maximally in the order of 3–4° (Table 2).

**Table 1:** Mean and standard deviation of the individual coefficient of multiple correlation (CMC) values, with and without correction for offset, for all rotations per carpal bone

CMC	Lunate	Scaphoid	Capitate
Raw			
Flexion-extension wrist			
*Flexion-extension*	0.99 ± 0.01	1 ± 0.003	–
*Radial-ulnar deviation*	0.99 ± 0.23	0.95 ± 0.08	0.97 (0.90–0.99)
*Pro-supination*	0.93 ± 0.11	0.92 ± 0.16	0.84 (0.42–0.95)
Radial-ulnar deviation wrist			
*Flexion-extension*	0.97 ± 0.02	0.94 ± 0.06	0.77 (NA*–0.86)
*Radial-ulnar deviation*	0.95 ± 0.09	0.93 ± 0.10	–
*Pro-supination*	0.84 ± 0.19	0.86 (0.71–0.96)	0.97 ± 0.03
Offset removed			
Flexion-extension wrist			
*Flexion-extension*	0.99 ± 0.01	1.00 ± 0.003	–
*Radial-ulnar deviation*	0.91 ± 0.21	0.96 ± 0.07	0.98 (0.95–0.99)
*Pro-supination*	0.95 ± 0.09	0.95 ± 0.08	0.86 (0.57–0.98)
Radial-ulnar deviation wrist			
*Flexion-extension*	0.98 ± 0.02	0.96 ± 0.04	0.86 (0.58–0.90)
*Radial-ulnar deviation*	0.96 ± 0.09	0.95 ± 0.09	–
*Pro-supination*	0.89 ± 0.154	0.94 (0.84–0.97)	0.98 ± 0.01

The median and interquartile range are reported when the CMC was not a real number for one or more test subjects

**Table 2:** Root mean square deviations (RMSD) of wrist kinematics between two repeated wrist motion cycles

	Lunate	Scaphoid	Capitate
Flexion-extension wrist			
*Flexion-extension*	3.16°	2.74°	–
*Radial-ulnar deviation*	1.51°	1.85°	3.01°
*Pro-supination*	1.17°	1.41°	3.19°
Radial-ulnar deviation wrist			
*Flexion-extension*	3,37°	4.29°	3.67°
*Radial-ulnar deviation*	1.62°	2.09°	–
*Pro-supination*	1.25°	1.73°	1.90°

## Discussion

In the present study, in vivo carpal kinematics of the scaphoid, lunate, and capitate in 20 healthy volunteers were assessed during flexion-extension and radial-ulnar deviation motion of the wrist with a dynamic 4D-CT imaging method. We found 4D-CT assessment of the wrist to be reliable, with overall mean and median coefficients of multiple correlations (CMCs) higher than 0.86 and RMSDs between 1.17° and 4.29°.

In the past, static images were used to detect morphologic differences, although joint motion is an essential part of diagnosing joint pathologies. A number of authors have described CT techniques to image and analyze the 3D kinematics of carpal bones. Most authors used in vitro models in which only one wrist was scanned [4, 5, 7]. Other authors used a step-wise motion of the wrist to reconstruct the total kinematics during full motion [17, 21, 22]. This stepwise technique provides an approximation of the true in vivo kinematics of the carpal bones. In wrists without any pathology, no significant differences were found between step-wise and dynamic techniques [8, 9]. However, in a pathological wrist, such as in individuals with a scapholunate ligament (SLL) injury, abrupt changes in kinematics can easily be missed.

In the last years, various dynamic imaging techniques enabled acquiring real-time functional images. Techniques such as sonography, MRI, and fluoroscopy are presented in the literature, however, all with specific advantages and limitations [23–26]. Sonography enables us to image joints and ligaments during activities, but bony structures cannot be visualized. Magnetic resonance imaging (MRI) provides good images of soft-tissue structures such as ligaments, but dynamic use is difficult as a result of low spatial and temporal resolution. Fluoroscopy provides dynamic image acquisition but produces images that show overlapping carpal bones when assessing a wrist. Therefore, CT images combined with 4D rotational X-ray was proposed as a method for dynamic imaging of the wrist joint [8, 9]. A drawback of this method is that it requires multiple cyclic motions of the hand to obtain 4D images, which might be a problem for patients and may introduce artifacts if the different motion cycles are not consistent. True 4D imaging with CT (i.e., 4D-CT) solves the abovementioned problems of other dynamic imaging techniques for the wrist joint.

There are several limitations to our study. First is that the sample our study was relatively small. However, it is thus far the largest group of uninjured in vivo wrists that has tried to establish a quantitative description of motion patterns and study test-retest reliability using the dynamic 4D CT method. Second, it was assumed that all wrists were uninjured and “normal” after medical history, physical examination, and Beighton scores were taken. Nevertheless, disorders or congenitally different shapes of the carpal bones that might affect the findings were not ruled out. Third, we did not study a possible relationship between carpal bone morphology and carpal kinematics. However, van de Giessen et al. [27] found that there is a wide range of lunate shapes instead of just five. Because of their findings and our small group of participants, it was decided not to investigate the influence of bone morphology on carpal kinematics.

Our study used an in vivo and dynamic CT technique in which a subject performed only one motion cycle. Repeated measurements to test test-retest reliability showed CMC values tending towards 1. This indicates that there is only a slight variability in measurements among participants. CMC values of the scaphoid and lunate kinematics were between 0.84 and 1. The CMC value of the capitate was slightly lower (lowest 0.77). However, clinically, the kinematics of the capitate are less important in patients with specific wrist pathology like scapholunate ligament injury. RMSDs were between 1.17° and 4.29°, which indicates that there is not much variability between the waveform of the first and repeated scan. For example, the lowest RMSD was 1.17° for the pro-supination of the lunate during flexion-extension of the wrist. The flexion-extension motion of the lunate, scaphoid, and capitate showed to have the largest variability in motion pattern during flexion-extension and radial-ulnar deviation of the wrist when comparing the first and repeated motion.

In conclusion, this study yields a unique data set of in vivo motion patterns in the wrist joint that describes the kinematics of the scaphoid, lunate, and capitate and its reliability in detail. This data set provides a valuable reference for future clinical studies with 4D-CT.

## Electronic supplementary material

Video 1 4D-CT Video of the radius, scaphoid, lunate and capitate while flexion-extension is performed. Upper left: transversal view, upper right: anterior coronal view, left bottom: radial sagittal view, right bottom: ulnar sagittal view. (AVI 137592 kb).

Video 2 4D-CT Video of the radius, scaphoid, lunate and capitate while radial-ulnar deviation is performed. Upper left: transversal view, upper right: anterior coronal view, left bottom: radial sagittal view, right bottom: ulnar sagittal view. (AVI 130623 kb).

ESM 1 (PNG 260 kb).

ESM 2 (PNG 286 kb).
