# Supplementary figures and images for: Quantifying in vivo scaphoid, lunate, and capitate kinematics using four-dimensional computed tomography
Source: Skeletal Radiol. 2020 Jul 30;50(2):351–9. doi: 10.1007/s00256-020-03543-4 (PMC7736028; doi:10.1007/s00256-020-03543-4)

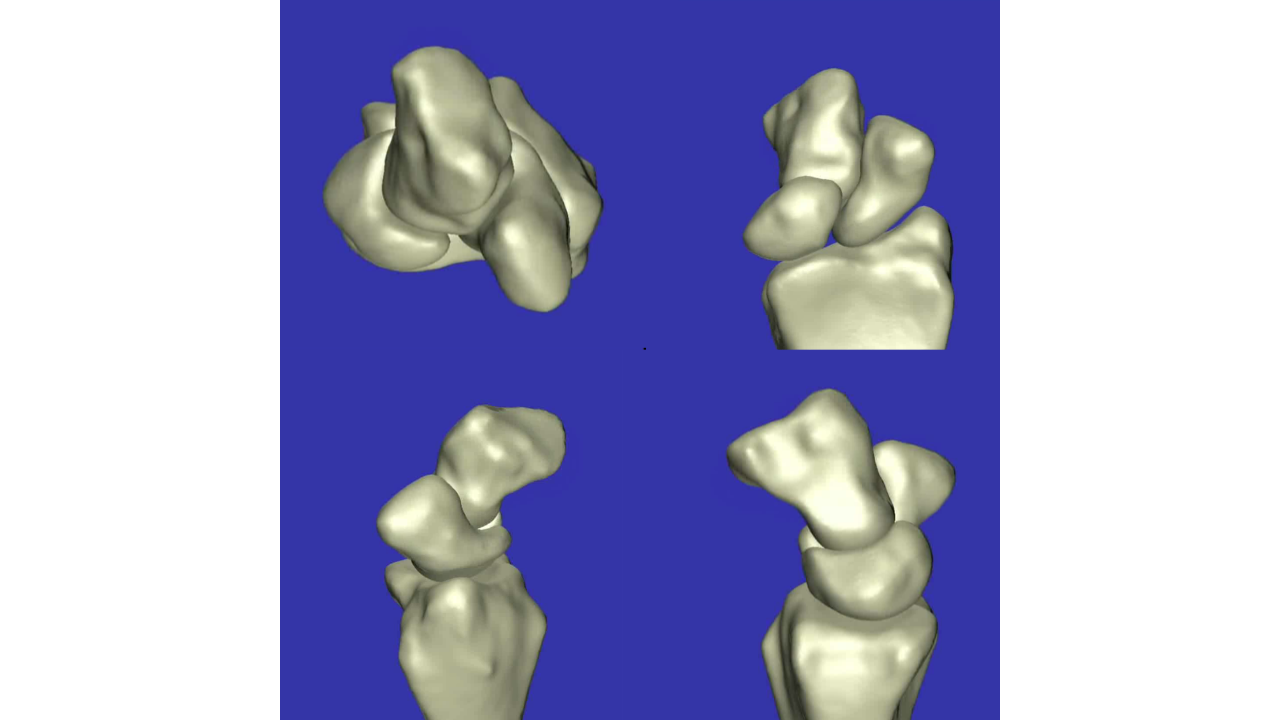

Supplement: Supplementary file 3 — (PNG 260 kb). [file 256_2020_3543_MOESM3_ESM.png]

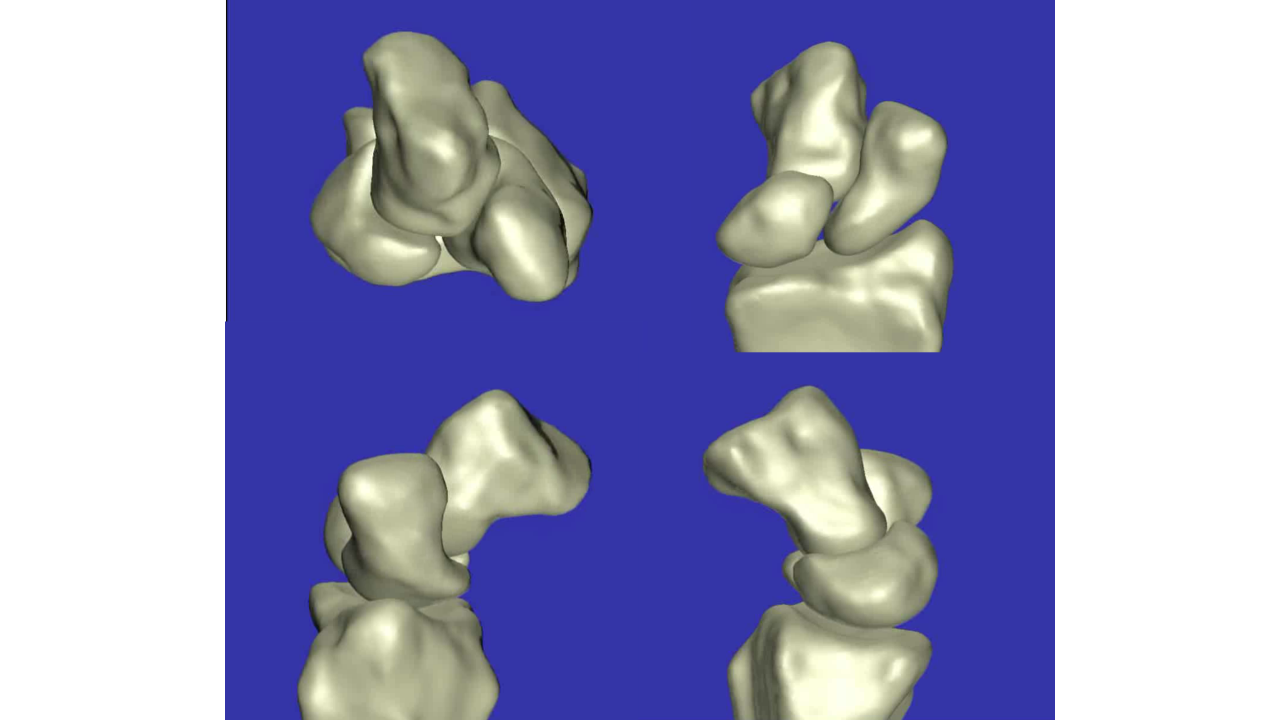

Supplement: Supplementary file 4 — (PNG 286 kb). [file 256_2020_3543_MOESM4_ESM.png]
